# Risk of allergic reactions to wine, in milk, egg and fish-allergic patients

**DOI:** 10.1186/2045-7022-1-10

**Published:** 2011-10-17

**Authors:** Emilia Vassilopoulou, Athanassios Karathanos, George Siragakis, Stavroula Giavi, Athanassios Sinaniotis, Nikolaos Douladiris, Montserrat Fernandez-Rivas, Michael Clausen, Nikolaos G Papadopoulos

**Affiliations:** 1 Allergy Research Centre, 2nd Department of Pediatrics, University of Athens, Greece; 2 Laboratory of Technology, Quality Control & Sensory Analysis of Wines & Spirits, Food Technology Dpt, Technological Educational Institute of Larissa, Karditsa, Greece; 3 NMR Laboratory, Department of Chemistry, University of Crete, 714 09 Iraklion, Crete, Greece; 4 Allergy Department, "Sotiria" General Hospital, Athens, Greece; 5 Allergy Service, Hospital Clinico San Carlos, Madrid, Spain; 6 Allergy Dpt. Landspitali University Hospital, Reykjavík, Iceland

**Keywords:** basophil activation, casein, fining agent, fish allergy, isinglass, milk allergy, questionnaire, skin prick test, wine

## Abstract

**Background:**

European legislators and wine producers still debate on the requirement for labeling of wines fined with potentially allergenic food proteins (casein, egg white or fish-derived isinglass). We investigated whether wines fined with known concentrations of these proteins have the potential to provoke clinical allergic reactions in relevant patients.

**Methods:**

In-house wines were produced for the study, fined with different concentrations of casein (n = 7), egg albumin (n = 1) and isinglass (n = 3). ELISA and PCR kits specific for the respective proteins were used to identify the fining agents. Skin prick tests and basophil activation tests were performed in patients with confirmed IgE-mediated relevant food allergies (n = 24). A wine consumption questionnaire and detailed history on possible reactions to wine was obtained in a multinational cohort of milk, egg or fish allergic patients (n = 53) and patients allergic to irrelevant foods as controls (n = 13).

**Results:**

Fining agents were not detectable in wines with the available laboratory methods. Nevertheless, positive skin prick test reactions and basophil activation to the relevant wines were observed in the majority of patients with allergy to milk, egg or fish, correlating with the concentration of the fining agent. Among patients consuming wine, reported reactions were few and mild and similar with the ones reported from the control group.

**Conclusion:**

Casein, isinglass or egg, remaining in traces in wine after fining, present a very low risk for the respective food allergic consumers. Physician and patient awareness campaigns may be more suitable than generalized labeling to address this issue, as the latter may have negative impact on both non-allergic and allergic consumers.

## Background

Wine production traditionally involves fining, during which some ingredients such as tannins are removed by co-precipitation with proteins derived from milk (casein, potassium caseinate), egg (ovalbumin, lysozyme) or fish (isinglass). Hypersensitivity reactions to wine have been reported rarely in the literature, attributed to grape proteins [1], biogenic amines, salicylates, sulfites or yeast [2,3]. No allergic reactions have been attributed to traces of fining agents in wine [4]; however, this possibility has not been ruled out. This has become more relevant with recent considerations on food labelling in Europe (2003/89/EC), Australia and the United States, requiring mandatory declaration on labels of wines, when substances that might provoke allergic reactions have been used in the production [5]. Although there was a provisional exclusion for all three fining agents from the label list no final decision is established [6], with additional special considerations about egg albumin fined wines [7].

Nevertheless, in-vivo evidence is scarce.

The aim of this study was to investigate whether wines fined with proteins derived from milk, egg or fish may have the potential to trigger allergic reactions in the respective food allergic patients.

## Methods

### Winery

Eleven wines were prepared for the needs of the study using standard winemaking. Seven wines were fined with different concentrations of casein, one with egg white and three with isinglass (Table 1). Wines 7 and 11 were purposefully fined with the highest usually used concentrations of casein and isinglass respectively [8].

**Table 1:** Wines produced for the study. Concentrations of the fining agents used correspond to the ones used in commercial wines apart from w7 and w11 where agents were used at the highest possible concentrations.

Code	Type of fining agent	Final concentration of fining agent
w1	control for casein fined wines	(-)

w2	Casein	120 mg/lt

w3	Casein	150 mg/lt

w4	Casein	200 mg/lt

w5	Casein	350 mg/lt

w6	Casein	450 mg/lt

w7	Casein	1000 mg/lt

***w8***	***control for egg & isinglass fined wines***	***(-)***

**w9**	**ovalbumin (egg white)**	**~50 mg/lt (~750 mg/lt)**

*w10*	*Isinglass*	*20 mg/lt*

*w11*	*Isinglass*	*60 mg/lt*

### Detection of fining agents in wine

ELISA tests were performed with wines containing casein and egg white using specific allergenic residue test kits in order to measure possible traces of the specific allergens (2.5-25 mg/l detection range) (Neogen Corporation, Scotland UK). A DNA based, real-time PCR was performed to detect traces of fish-derived genetic material in isinglass-fined wines (detection level 10 copies) (CONGEN Biotechnology GmbH, Berlin Germany).

### Patients

Twenty four individuals (13 male; age range: 2-36 years, mean 11.63 ± 6.77 years) with IgE-mediated food allergy, diagnosed within the last year by open or double-blind challenge and a positive skin prick and/or CAP FEIA tests to at least one of milk (casein), egg or fish participated in the study. Eleven were allergic to milk (CAP to casein = 7.12 ± 6.59 kU/l), 7 to egg (CAP to egg white = 8.22 ± 4.97 kU/l) and 6 to fish (CAP to cod = 17.56 ± 31.42 kU/l).

In order to exclude non-specific in-vivo or in-vitro reactions to wines, a control group of comparable age and sex distribution was included in the study, constituted by atopic, food allergic patients reacting to other foods (n = 8), or healthy, non atopic, non food allergic individuals (n = 5). The study was approved by the local ethics committee and informed consent was provided by participants or their guardians.

### Wine consumption survey

A simple, wine consumption frequency questionnaire was performed in 53 adult patients (27 male, 35 ± 14 years) from Greece (n = 36), Iceland (n = 9) and Spain (n = 8) with diagnosed food allergy in one of the three offending fining agents (41 to fish, 7 to egg and 5 to milk). The inclusion criteria and the questionnaire on wine consumption are provided in the appendix. Twelve patients (4 male, 38 ± 13.86 years) with other food allergies (12 shrimp, 3 apple and 1 peanut allergy) were used as controls. Consumption of wines with known fining agents (according to the manufacturers) was specifically queried (Additional file 1).

### Skin prick Testing

Skin prick testing with commercial extracts and relevant wines were performed according to standard protocols [9,10]. Any size of wheal larger than the negative control was considered positive.

### Basophil activation test

Basophil activation was determined with Basotest (Orpegen Pharma, Germany) in heparinized whole blood samples, according to the protocol of the manufacturer. Cells were incubated with the relevant wines after dialysis against PBS (white wines) or precipitation with ethanol and resuspension in PBS to the original volume of the wine [4].

### Statistical analysis

Statistical analysis was conducted with SPSS software. Continuous variables were assessed for normality and log-transformed where appropriate. Comparison between groups was performed using repeated measures analysis of variance. Pearson analysis was used to examine categorical data by chi-square tests. P values < 0.05 were considered statistically significant.

## Results

### Allergen detection in wines

Even though concentrations of fining agents up to the usually permitted were added to wines, no allergens were detectable after the fining process in any of the wines tested, with either ELISA or PCR.

### Skin reactivity

There was no skin reaction to any of the wines in the control population. Among 11 milk allergic patients, 91% had a positive reaction to at least one casein containing wine. The proportion of patients reacting to casein-fined wines generally increased with increasing concentration of fining agent used (w1 (control) = 0, w2 = 18%, w3 = 9%, w4 = 18%, w5 = 27%, w6 = 36%, w7 = 73%). In comparison to control wine (w1, no positive skin reactions), the average size of wheals was significantly higher for w6 (mean ± STDV: 1.5 mm ± 2.3, p = 0.051) and w7 (3.2 mm ± 2.3, p = 0.02). Only 1/7 (14%) egg allergic patients had a positive SPT with the respective wine fined with egg white (w9). Four out of 6 (66%) fish-allergic patients reacted to both low and high isinglass containing wines (w10 and w11), whereas none reacted to the respective control wine (w8).

### Basophil activation

A small, but significant induction of basophil activation was observed with the high concentration casein-fined wine extract (w7) (mean ± STDV: 14.9% ± 1.7), in comparison to the control w1 (11.3% ± 2.1) (p = 0.015), in patients with allergy to milk. This was not the case for the low concentration casein fined wine (w2) (12% ± 2.8). Significant activation was also observed in basophils from egg and fish allergic patients respectively with w9 (egg white-fined) (10.3% ± 2.9, p = 0.018) and w10 (isinglass fined) (13.3% ± 7.5, p = 0.02), in comparison to the control w8 (8.2% ± 7).

In all cases, the percentage of activated basophils in positive controls (anti-IgE, fMLP, and/or relevant allergen extract) was >20%, thus validating each assay [11]. No significant basophil activation was observed in control allergic and non-allergic individuals (not shown).

### History of wine consumption

Forty seven out of 54 milk, egg or fish allergic patients consumed wine without a problem with an average frequency of 1.5 times per week (mean ± STDV: 1/day - 1/year; stdv ± 1.5 times per week) in average quantities of 2 glasses/time (1 glass to 1 liter per time; ± 1 glass). Minor complaints, potentially associated with allergy were mentioned by 4 patients, (2 fish and 2 egg allergic) after wine drinking (4 itching, 1 runny or stuffy nose, 1 cough, 1headache). One patient with fish allergy and a history of anaphylaxis attributed to beer, usually fined with isinglass, consumed 4 glasses of wine 3 times per week without a problem.

Nevertheless, 6 out of 12 control patients also reported minor symptoms after consuming wine (1 itching, 3 rash, 1 runny or stuffy nose, 5 headaches).

## Discussion

No detectable traces of allergenic proteins used in fining could be found in experimentally produced wines by sensitive in-vitro methods, in agreement with a previous investigation in commercial wines [12].

However, sub-trace amounts of milk, fish and egg allergens are still able to elicit IgE-mediated skin responses and in-vitro basophil activation in sensitized patients. The magnitude of the responses was quite low; however, this should be expected from minute allergen concentrations. The higher sensitivity of the skin in comparison to in-vitro methods has also been observed in other settings [9]. Another study by Kirschner et al, wines containing concentrated fining agents were allergenic in skin prick tests, but provocation tests with these fined wines were negative [13].

Although no reactions to wine attributable to fining agents have been reported so far, concerns on such potential should be taken into account. Individual proteins should be addressed separately. A recent proposal by the European Food Safety Authority pointed out egg protein traces for special consideration [7]. This is not supported by our findings, as the egg-fined wine, at a concentration used in real life, had the lowest proportion of skin reactivity. Furthermore egg allergy is rather rare in adults [14]. This is even more so for milk allergy which is almost always a pediatric problem [15]. Thresholds for allergic reactions to these proteins have not been conclusively established; however in a recent report the defined risk (p ≥ 0.5) for allergic reaction in adults was for a cumulative dose of 1000 mg to milk, 90 mg to fish and 0.012 mg to egg [16].

Adult prevalence of food allergy can be up to 2% of the population [17], with fish accounting for a proportion of anaphylactic reactions sometimes in very low thresholds [18]. Nevertheless, the resulting danger for potential reactions is probably negligible. This is supported by the fact that patients with diagnosed fish allergy and a positive SPT to isinglass-fined wines were still able to consume moderate amounts of wine without any problem, as resulted from the current study. A recent study was able to detect allergens in wine using highly sensitive methodology, without however being able to induce a clinical reaction in mice sensitized to ovalbumin, caseinate, or isinglass [19].

Protecting allergic consumers from accidental exposure to hidden allergens is of paramount importance for the quality of life of this increasing population. However, excessive usage of warning labels restricts consumer choice leading to strict, sometimes unnecessary avoidance of foods. On the other hand the impact of such warnings can be devalued with parallel increased risk of unintentional consumption.

In conclusion, current evidence indicates a very low, if existent, risk for the allergic consumer from wine-fining agents [20]. Promoting awareness of specialized health care professionals and through them to susceptible individuals may be able to prevent potential reactions in extremely sensitive individuals, without compromising the needs of the general population.

## Competing interests

The authors declare that they have no competing interests.

## Authors' contributions

EV organized and supervised the whole study, performed ELISA tests and Basotests, made the statistical analysis and drafted the manuscript. AK did the winemaking. GS did the quantitative analysis of suspicious proteins in wines with PCR and provided specific ELISA tests for egg, milk, fish quantitation. SG, AS, ND, MFR, MG, and NGP were responsible for collecting clinical data for all different patients included in the study and perform in vivo tests or fulfill questionnaires. NGP supervised the study and contributed in drafting. All authors read and approved the final manuscript.

## Supplementary Material

Additional file 1 Survey on wine consumption of milk, egg and fish allergic patients Click here for file
