# Supplementary material for: Risk of allergic reactions to wine, in milk, egg and fish-allergic patients
Source: Clin Transl Allergy. 2011 Oct 17;1:10. doi: 10.1186/2045-7022-1-10 (PMC3339366; doi:10.1186/2045-7022-1-10)
Supplement: Additional file 1 — Survey on wine consumption of milk, egg and fish allergic patients [file 2045-7022-1-10-S1.RTF]

Additional File 1
Survey on wine consumption of milk, egg and fish allergic patients

Inclusion criteria

 >18 years of age 

Cases: with a confirmed diagnosis (according to the centre's practices) of IgE-mediated allergy to milk and/or egg, and/ or fish, active during the last 24 months 

Controls: with a confirmed diagnosis (according to the centre's practices) of IgE-mediated allergy to allergens other than milk, egg or fish, not excluded below 

Who have consumed wine during the same period 

Exclusion criteria

grape allergy 
gluten and or wheat allergy 
yeast allergy 


Please provide the following data from patient's clinical history

Sex (M/F)		
Age (years)		
Offending food(s)		

Per food:
Time from consumption to reaction (min) 	
	
Severity of the most severe reaction (mild, moderate, severe anaphylaxis)	
	
CAP kU/L  (if available)		
SPT max wheal diameter (if available)		


 Questionnaire on wine consumption

1)	Do you consume wine?: Yes  No
2)	If Yes how often? (per day, week, month or year)
3)	Can you define an average portion you drink? (eg 2 glasses each time)
4)	Please define the wine type(s) (white, red or a label you have consumed several times)
5)	Did you ever have any of the following symptoms after drinking wine in moderate quantity? 
	Yes 	No	
Itching, tingling or swelling in the mouth, lips or throat			
A rash, nettle sting like rash or itchy skin			
Runny or stuffy nose. Red, itchy or running eyes			
Difficulty swallowing			
Cough, wheeze or breathlessness			
Diarrhoea or vomiting			
Fainting or dizziness			
Headaches			
